# Correction: Cigarette smoking and associated factors among men in five South Asian countries: A pooled analysis of nationally representative surveys

**DOI:** 10.1371/journal.pone.0315447

**Published:** 2024-12-05

**Authors:** Md Shariful Islam, Mamunur Rashid, Monaemul Islam Sizear, Raafat Hassan, Mahbubur Rahman, Sarker Masud Parvez, Shuvon Chandra Hore, Rehnuma Haque, Farjana Jahan, Supta Chowdhury, Tarique Mohammad Nurul Huda, K. M. Saif-Ur-Rahman, Arifuzzaman Khan

The affiliation for the sixth author is incorrect. Sarker Masud Parvez is not affiliated with #10 but with #9: Faculty of Medicine, The University of Queensland, Brisbane, Queensland, Australia.
