# Supplementary material for: Cigarette smoking and associated factors among men in five South Asian countries: A pooled analysis of nationally representative surveys
Source: PLoS One. 2022 Nov 14;17(11):e0277758. doi: 10.1371/journal.pone.0277758 (PMC9662728; doi:10.1371/journal.pone.0277758)
Supplement: S1 Table — (DOCX) [file pone.0277758.s001.docx]

| **Potential predictors** | **Questions and responses** [1-5] |
| --- | --- |
| Age | How old were you on your last birthday? (response taken in completed years) |
| Marital status | What is your current marital status? (Currently married=1, married/ gauna not performed= 2, Widowed= 3, Divorced= 4, Separated= 5, Deserted= 6, Never married= 7 |
| Place of residence | Type of primary Sampling Unit (Urban = 1, Rural = 2) |
| Region | State/Province of residence |
| Education status | What is the highest standard you completed? (response taken in completed standard number) |
| Occupation | What is your occupation, that is, what kind of work do you mainly do? (did not work= 0, professional/technical/managerial= 1, clerical= 2, sales= 3, agricultural – self-employed= 4, agricultural – employee= 5, household and domestic= 6, services=7, skilled manual=8, unskilled manual= 9, armed forces= 10) |
| Wealth status of household | Implementing partners in each country classified household wealth status based on household asset and dwelling characteristics, such as a car and a television; housing conditions such as flooring content, water supply, toilet facilities; and other wealth-related factors. For each item, a standardized score is allocated to each household, with the score varying based on whether the household owns the asset or not. These scores are added together by household. Respondents are ranked according to their household's overall score. The survey is then grouped into population quintiles — five classes of the same size and the same number of people in each quintile and classify the wealth quintiles as poorest (1), poorer (2), middle (3), richer (4), and richest (5) [6]. |
| Reading newspaper or magazine | Do you read a newspaper or magazine almost every day, at least once a week, less than once a week, or not at all? ( Almost every day =1, At least once a week= 2, Less than once a week= 3, Not at all=4) |
| Frequency of watching television | Do you watch television almost every day, at least once a week, less than once a week or not at all? ( Almost every day =1, At least once a week= 2, Less than once a week= 3, Not at all=4) |
| Frequency of listening to the radio | Do you listen to the radio almost every day, at least once a week, less than once a week or not at all? ( Almost every day =1, At least once a week= 2, Less than once a week= 3, Not at all=4) |

**S1 Table. Potential predictors of cigarette smoking among men in the South Asian countries in DHS and NHFS survey, 2015-2018**

# References

1. Central Statistics Organization (CSO), Ministry of Public Health (MoPH), and I. *Afghanistan Demographic and Health Survey 2015.*; Kabul, Afghanistan: Central Statistics Organization., 2017;

2. Ministry of Health, N.N.E. and I. *Nepal Demographic and Health Survey 2016.*; Kathmandu, Nepal: Ministry of Health, Nepal, 2017;

3. ICF., N.I. of P.S. (NIPS) [Pakistan] and *Pakistan Demographic and Health Survey 2017-18.*; Islamabad, Pakistan, and Rockville, Maryland, USA: NIPS and ICF., 2019;

4. International Institute for Population Sciences *National Family Health Survey (NFHS-4), 2015-16: India. Mumbai:: International Institute for Population Science and ICF*; 2017;

5. Rao, S.; Aslam, S.K.; Zaheer, S.; Shafique, K. Anti-smoking initiatives and current smoking among 19,643 adolescents in South Asia: findings from the Global Youth Tobacco Survey. *Harm Reduct. J.* **2014**, *11*, 1–7, doi:10.1186/1477-7517-11-8.

6. Rutstein, S.O.; Johnson, K. The DHS wealth index. DHS comparative reports no. 6. *Calvert. ORC Macro* **2004**.
