# Supplementary material for: Cigarette smoking and associated factors among men in five South Asian countries: A pooled analysis of nationally representative surveys
Source: PLoS One. 2022 Nov 14;17(11):e0277758. doi: 10.1371/journal.pone.0277758 (PMC9662728; doi:10.1371/journal.pone.0277758)
Supplement: S2 Table — (DOCX) [file pone.0277758.s002.docx]

| **Country** | **Prevalence %(95% CI)** |
| --- | --- |
| Afghanistan | 22.0 (20.6 - 23.5) |
| India | 23.0 (22.5 - 23.5) |
| Maldives | 41.2 (39.2 -43.2) |
| Nepal | 26.9 (24.3 - 29.7) |
| Pakistan | 20.1 (17.9 - 22.5) |
| ^a^ Data were from the standard Demographic and Health Survey (DHS) conducted among men aged15–49 years old in India in 2016, Nepal in 2016, Pakistan in 2018, the Maldives in 2017, and Afghanistan in 2015. A complex survey design and sampling weight were applied. Prevalence with 95% Confidence Interval in parentheses is shown. A chi-square test was performed to calculate the *p*-value | |

**S2 Table. Country-wise prevalence of cigarettes smoking among men in the South Asia^a^**
