# Supplementary material for: Cigarette smoking and associated factors among men in five South Asian countries: A pooled analysis of nationally representative surveys
Source: PLoS One. 2022 Nov 14;17(11):e0277758. doi: 10.1371/journal.pone.0277758 (PMC9662728; doi:10.1371/journal.pone.0277758)
Supplement: S3 Table — (DOCX) [file pone.0277758.s003.docx]

| **Afghanistan** | | | | | | | |
| --- | --- | --- | --- | --- | --- | --- | --- |
| Region | Estimate | Region | Estimate | Region | Estimate | Region | Estimate |
| Kabul | 24.6 | Baghlan | 15.9 | Badakhshan | 4 | Urozgan | 25 |
| Kapisa | 27.2 | Bamyan | 7 | Takhar | 8.3 | Zabul | 28.7 |
| Parwan | 28.4 | Ghazni | 25.8 | Kunduz | 30.3 | Kandahar | 33.5 |
| Wardak | 18.1 | Paktika | 11.1 | Samangan | 8.3 | Jawzjan | 49.5 |
| Logar | 19.7 | Paktya | 14 | Balkh | 16 | Faryab | 22.3 |
| Nangarhar | 23.6 | Khost | 16.5 | Sar-E-Pul | 14.6 | Helmand | 19.7 |
| Laghman | 23.2 | Kunarha | 5.5 | Ghor | 20.2 | Badghis | 13.1 |
| Panjsher | 28.5 | Nooristan | 27.6 | Daykundi | 2.6 | Herat | 25.8 |
| Farah | 37.2 | Nimroz | 2.5 |  |  |  |  |
| **India** | | | | | | | |
| Region | Estimate | Region | Estimate | Region | Estimate | Region | Estimate |
| Andaman & nicobar islands | 16 | Daman and Diu | 12.2 | Madhya Pradesh | 28.5 | Himachal Pradesh | 35.4 |
| Andhra Pradesh | 20.9 | Arunachal Pradesh | 38.9 | Dadra and Nagar Haveli | 15.8 | Jammu & Kashmir | 33.2 |
| Assam | 33 | Haryana | 31 | Manipur | 42.1 | Sikkim | 28.6 |
| Chandigarh | 20 | Punjab | 14.1 | Meghalaya | 64.3 | Tamil Nadu | 27.3 |
| Chhattisgarh | 17.4 | Maharashtra | 10.7 | Mizoram | 72.1 | Tripura | 50.2 |
| Bihar | 17.5 | Jharkhand | 15.3 | Nagaland | 34.4 | Uttar Pradesh | 24.9 |
| Gujarat | 12.8 | Karnataka | 22.5 | New Delhi | 20.4 | Uttarakhand | 29.8 |
| Goa | 12.8 | Kerala | 22.5 | Odisha | 18.7 | West Bengal | 42.5 |
| Rajasthan | 24.6 | Lakshadweep | 22.9 | Puducherry | 12.4 | Telangana | 18.3 |
| **Maldives** | | | | | | | |
| Region | Estimate | Region | Estimate | Region | Estimate | Region | Estimate |
| Male | 42 | North Central | 44.1 | South Central | 39.1 | South | 40.6 |
| North | 34.6 | Central | 45.8 |  |  |  |  |
| **Nepal** | | | | | | | |
| Region | Estimate | Region | Estimate | Region | Estimate | Region | Estimate |
| Province 1 | 26.5 | Province 3 | 30.9 | Province 5 | 22 | Province 7 | 39.5 |
| Province 2 | 21.7 | Province 4 | 24.6 | Province 6 | 29 |  |  |
| **Pakistan** | | | | | | | |
| Region | Estimate | Region | Estimate | Region | Estimate | Region | Estimate |
| Punjab excluding Islamabad (ICT) | 24.5 | Khyber Pakhtunkhwa | 11.8 | Islamabad (ICT) | 26.6 | Federally Administered Tribal Areas | 11.3 |
| Sindh | 16.3 | Balochistan | 18.4 |  |  |  |  |
| ^a^ Data were from a standard Demographic and Health Survey (DHS) conducted among men aged 15–49 years old in India in 2016, Nepal in 2016, Pakistan in 2018, the Maldives in 2017, and Afghanistan in 2015. A complex survey design and sampling weight were applied. A chi-square test was performed to calculate the *p*-value. | | | | | | | |

**S3 Table. State-wise prevalence of cigarettes smoking among men in the South Asia^a^**
