# Supplementary material for: Cigarette smoking and associated factors among men in five South Asian countries: A pooled analysis of nationally representative surveys
Source: PLoS One. 2022 Nov 14;17(11):e0277758. doi: 10.1371/journal.pone.0277758 (PMC9662728; doi:10.1371/journal.pone.0277758)
Supplement: Attachment — Submitted filename: Reviewer Comments.docx [file pone.0277758.s004.docx]

Reviewer Comments

Reviewer 1

The manuscript well written and easy to be followed
The section about public health implication needs to be expanded and provide specific country specific implication
The title of the figure should be under not above it.
Some figures have no titles

Reviewer 2

The study is apt and stands to contribute the the existing body of knowledge. It is however important that following revision are made

Introduction
1. Region in line 59 should be regions
2. The sentence on lines 59 - 61 should be revised particularly "The World Health Organization (WHO) set" is not clear

Methods
1. It appears that sample size for this study was not estimated which could have provided the basis for generalization and drawing of statistical inference. It will be good if the authors can provide the needed information in this regard in as much this study had used secondary data abstraction.
2. The rationale for using two different data sources (DHS and NHFS) is not clear and it will be good if a follow up information on this is provided in the manuscript. Additionally since these selected enumeration areas have different number of households, it would have been more scientific to have used proportion to size technique to determine number of households to be selected rather than picking fixed number of households per enumeration areas. However, provision of the rationale for the use of fixed number households by the authors will suffice.
3. The authors will need to provide more clarification of what the secondary data abstraction from DHS and NHFS were used for and that of the face to face interview. Additionally, the source of data collection tool used for face to face
interview should provided as well as how the reliability and validity of the tool were ascertained?
4. The authors have not included the criteria for inclusion in the study. It will be good if the inclusion and exclusion criteria used are clear stated. Additionally, the study focused on males only but females also engage in tobacco use, the rationale for excluding female may be needed.
5. The authors had stated that face to face interviews were conducted, however, it is unclear what that was used to achieve. Additionally, since there is a face to face component of the data collection, it is important that details of how consent for participation was obtained and also ethical approval may be required as tis is different from the DHS

Results
1.It is unclear how NA was taken care off or adjusted for in the analysis on table 2 and 3?
