# Supplementary material for: Cigarette smoking and associated factors among men in five South Asian countries: A pooled analysis of nationally representative surveys
Source: PLoS One. 2022 Nov 14;17(11):e0277758. doi: 10.1371/journal.pone.0277758 (PMC9662728; doi:10.1371/journal.pone.0277758)
Supplement: Attachment — Submitted filename: Reviewer Comments.docx [file pone.0277758.s005.docx]

**Response to Reviewers**

Thank you for reviewing the manuscript critically. Your comments are valuable to improving the quality of the manuscript. We have updated the manuscript based on your comments.

**Reviewer 1**

The manuscript well written and easy to be followed
The section about public health implication needs to be expanded and provide specific country specific implication

**Response:** Thank you for your compliments.

The title of the figure should be under not above it.
Some figures have no titles

**Response:** Thank you for this comment. We replace the title of Table 1,2,3 from above to the bottom of the tables. Please see at page #8 line #171, page #13 line #203, and page #17 line #258. We also added the title of Figures 1, 2,3, and 4. Please see pages #32-35. We also replace the position of title in the supplementary file. Please see pages #1,2 and 3.

**Reviewer 2**

The study is apt and stands to contribute the the existing body of knowledge. It is however important that following revision are made

**Introduction**
1. Region in line 59 should be regions

**Response:** Change has been made accordingly, see in line #56.

2. The sentence on lines 59 - 61 should be revised particularly "The World Health Organization (WHO) set" is not clear

**Response:** Thank you for your comments. We have updated the sentence. Please see lines 58-60. We removed the word "set ". It seems redundant. Now, the sentence is " The World Health Organization (WHO) introduced a target of a 25% reduction of death among individuals aged 30–70 years from cardiovascular diseases, diabetes, cancer, and chronic respiratory diseases between 2010 and 2025 ".

**Methods**
1. It appears that sample size for this study was not estimated which could have provided the basis for generalisation and drawing of statistical inference. It will be good if the authors can provide the needed information in this regard in as much this study had used secondary data abstraction.

**Response:** Thank you for your insightful comment. The datasets we merged were national representative for urban and rural areas, and for the first administrative level subdivisions, district data, and the response rate was quite high for each country (approximately 92% on average). The DHS research team calculated the sample size. Sample sizes for these DHS surveys are based on the number of survey domains (usually subnational units such as regions).. All survey sampling strategies are subject to sampling error. The DHS Program designs samples to provide national and subnational estimates with a reasonable relative standard error. We included all samples in our data analysis. As the data were represented nationally, even representative for the first administrative level, we think that there is no problem with the external validity (generalizability) of this study. Moreover, the high response rate and adequate sample size of each country provided sufficient statistical power, which abetted us to draw statistical inferences right. This has been clarify in the Method section page#4-5, lines#96-101, and in Discussion page#19, lines# 327-330.

2. The rationale for using two different data sources (DHS and NHFS) is not clear and it will be good if a follow up information on this is provided in the manuscript. Additionally since these selected enumeration areas have different number of households, it would have been more scientific to have used proportion to size technique to determine number of households to be selected rather than picking fixed number of households per enumeration areas. However, provision of the rationale for the use of fixed number households by the authors will suffice.

**Response:** In India, DHS is known as NHFS. NHFS is the same as DHS. We have now updated line #86. About picking a fixed number of households per enumeration area, we agree with you that using the proportion to size technique to determine the number of households could be a better option. However, DHS picked a fixed number of households. The explanation from DHS is that a fixed number of households was selected to avoid the logistical burdens caused when the variable number of households is selected. At the same time, it is more cost-efficient.

3. The authors will need to provide more clarification of what the secondary data abstraction from DHS and NHFS were used for and that of the face to face interview. Additionally, the source of data collection tool used for face to face interview should provided as well as how the reliability and validity of the tool were ascertained?

**Response:** We did not conduct any interviews. DHS surveys collect data through face-to-face interviews using households questionnaire incorporating reliable and valid tools and instruments. We’ve now revised our text related to a face-to-face interview in the manuscript, please see page #4, lines #99 – 100.

4. The authors have not included the criteria for inclusion in the study. It will be good if the inclusion and exclusion criteria used are clear stated. Additionally, the study focused on males only but females also engage in tobacco use, the rationale for excluding female may be needed.

**Response:** We revised and added inclusion and exclusion criteria on page 5, lines #104-107. We excluded women because the prevalence of cigarette smoking among women in South Asian countries is very low. In South Asia, women use mainly smoke-less tobacco, which is out of interest in this study.

5. The authors had stated that face to face interviews were conducted, however, it is unclear what that was used to achieve. Additionally, since there is a face to face component of the data collection, it is important that details of how consent for participation was obtained and also ethical approval may be required as tis is different from the DHS.

**Response:** We did not conduct any interviews. We only analyse data from DHS. We think we were not clear enough hence the confusion. We revised our writing about face-to-face interviews. Please see page #5, lines #99, and 100. The DHS program took ethical approval to conduct surveys in Afghanistan, Nepal, India, Pakistan, and the Maldives. The DHS program also took written consent to collect data from participants. We took approval from the DHS program to perform this data analysis. Please see page#7, line #155-156.
**Results**
1.It is unclear how NA was taken care off or adjusted for in the analysis on table 2 and 3?

**Response:** Among the five South-Asian countries we analysed data, the DHS in Afghanistan and Pakistan did not include men who are never married. Those countries included populations who were married or formally married. For this reason, the option became not applicable for these two countries. In our analysis, we used the currently married option as a reference value which is available for all countries. In our pool analysis, we re-normalised the sampling weight.
